# Implementing a health-system–wide antibiotic stewardship program in ambulatory surgery centers

**DOI:** 10.1017/ash.2023.250

**Published:** 2023-09-29

**Authors:** Kasey Hickman, Nicolas Forcade, Mandelin Cooper, Shivanne Bhagwandeen, Brandy Russell

## Abstract

**Background:** In 2016, the CDC released the Core Elements of Outpatient Antibiotic Stewardship, which extended the requirements previously released for hospital facilities and nursing homes to the outpatient setting. Several regulatory agencies focused on outpatient antimicrobial use. However, The Joint Commission and the Ambulatory Surgery Center (ASC) Leapfrog Group excluded ambulatory surgery centers from their medication management standards and questions. Due to the public health and patient safety benefits of implementing an antimicrobial stewardship program (ASP) and increasing regulatory interest in the matter, the Hospital Corporation of America (HCA) Ambulatory Surgery Division formally launched a nationwide ASP for its ambulatory surgery centers in March 2021. **Methods:** HCA is a large healthcare system with 146 ASCs in 16 states in 2021. The structure of the ASCs are local surgery centers with a medical director, a nurse responsible for infection prevention, and a pharmacist at a regional level. The types of surgeries vary based on location and ASC site. In 2019, a multidisciplinary team formed the corporate planning committee. The program was modeled after the CDC Core Elements and The Joint Commission’s requirements for an ASP. Each ASC was asked to build a local ASP team, led by a local physician and a regionally based pharmacist. In addition, a stewardship goal was established to update all preoperative antibiotic surgical-site infection prophylaxis order sets. The corporate committee provided educational resources, including evidence-based guidelines for appropriate antibiotic selection for surgical-site infections. They collected antibiotic cost per case as a baseline metric to track and analyze. Pediatric, ophthalmic, and gastrointestinal endoscopic procedures were excluded from the program. **Results:** From January 1, 2020, through December 31, 2021, including only centers that were operational during this period and excluding single specialty endoscopy centers, antibiotic cost per case decreased annually from $2.38 to $1.84 (*t* = 4.157; *P* < .005), and the postoperative infection rate also declined from 0.370 to 0.304 (*t* = 2.079; *P* = .040). **Conclusions:** Our findings suggest that implementing a health-system–wide outpatient antibiotic stewardship program in the ambulatory surgery center setting is feasible and may contribute to decreased antibiotic cost per case and improved postoperative surgical site infection rates.

**Disclosures:** None

